# Elevated Expression of Cytosolic Phospholipase A2Delta Is Associated with Lipid Metabolism Dysregulation during Hepatocellular Carcinoma Progression

**DOI:** 10.22074/cellj.2020.6527

**Published:** 2019-09-08

**Authors:** Maryam Ranjpour, Saima Wajid, Swatantra Kumar Jain

**Affiliations:** 1 Department of Biotechnology, School of Chemical and Life Sciences, Jamia Hamdard, New Delhi, India; 2 Department of Medical Biochemistry, HIMSR, Jamia Hamdard, New Delhi, India

**Keywords:** Chemical Carcinogens, Cytosolic Phospholipase A2Delta, Hepatocellular Carcinoma, MALDI-TOF-MS/MS, Western Blot Analysis

## Abstract

**Objective:**

Liver cancer is the third rank amongst the common malignancies, causing maximum death in the patients diagnosed with cancers. Currently available biomarkers are not enough sensitive for early diagnosis of hepatocellular carcinoma (HCC). This makes difficult management of HCC. With the aim of finding new generation of proteomic-based biomarkers, the represented study was designed to characterize the differentially expressed proteins at different stages of HCC initiation and at progression. This could lead to find potential biomarkers for early detection of HCC.

**Materials and Methods:**

In this experimental study, we report induction of HCC by administrating chemical carcinogens in male Wistar rats. Disease progression was monitored by histological evaluation. Serum proteomic analyses such as 2 dimensional (2D)-electrophoresis, MALDI-TOF-MS/MS and Western blot have been used to analyze and characterize the differentially expressed proteins during HCC development.

**Results:**

HCC initiation and tumorigenesis were observed at one and four months post carcinogen treatment, respectively. One of the differentially-expressed proteins, namely, cytosolic phospholipase A2delta was significantly up-regulated at very early stage of HCC development. Its expression continued to increase during cancer progression and hepatotumorigenesis stages. Its elevated expression has been confirmed by Western blot analysis. Consistent to this, analyses of the sera in the clinically confirmed liver cancer patients showed elevated expression of this protein, further validating our experimental results.

**Conclusion:**

This study suggests that elevation in the expression of cytosolic phospholipase A2delta is associated with progression of HCC.

## Introduction

Hepatocellular carcinoma (HCC) is globally the fifth most common cancer with a high rate of morbidity and the third type of cancer causing maximum death among the patients diagnosed with cancers (1, 2). Etiological influences such as hepatitis B virus (HBV) and hepatitis C virus (HCV) infections, alcohol abuse, metabolic diseases and carcinogen exposure lead to chronic inflammation of liver and mutation causing heterogeneous HCC (3). Lack of clear symptoms, numerous relapse and inefficient therapy lead to poor prognosis and high mortality in patients diagnosed with HCC (2). Finding new generation noninvasive biomarkers to detect HCC at early stage would help reduce the rate of cancer-related mortality (4). Currently available markers, such as alpha-fetoprotein, do not have high sensitivity and search for novel markers is mandatory. Effective treatment and patient survival rate are dependent on the early diagnosis of HCC which can be provided based on the novel prognostic and diagnostic biomarkers (5). 

In the present study, using animal model, we aimed to find out differentially expressed proteins that are associated with HCC initiation and progression to introduce as potential biomarker(s) or to target as therapeutic agent at very early stage of liver cancer initiation.

## Materials and Methods

The experimental study involves analysis of rodent model *in vivo* which has previously been developed in our laboratory to study HCC. Further, the obtained data are validated with sera of clinically approved liver cancer patients. 

### Liver cancer induction and development of the rodent model

Liver cancer was chemically induced in 4-6 weeks old male Wistar rats weighing 80-100 g, by administrating chemical carcinogens DEN and 2-AAF as reported by our group earlier. Animal experimentation was performed following approval from Jamia Hamdard (New Delhi, India) Institutional Animal Ethics Committee formed for the Purpose of Control and Supervision of Experiments on Animals (project number 908). The protocol for HCC development in rats was essentially the same as previously described instruction (6). Briefly, the rats were kept in polypropylene cages while temperature was maintained at 25 ± 2°C with 12 hours cycle of light/dark in the animal house of Jamia Hamdard. These were fed ad libitum with free access to standard laboratory food (Amrut Laboratory, rat and mice feed, Navmaharashtra Chakan Oil Mills Ltd., India) and water daily. DEN (200 mg/kg body weight) and 2-AAF dissolved in 1% carboxymethyl cellulose (150 mg/kg body weight) were used as the initiator and promoter of HCC, respectively. Animals were randomly split up into two groups namely control and treated groups. Treated groups were further divided into two different groups namely, 1 M (sacrificed after one month) and 4 M (sacrificed after four months). The carcinogen treated animals were given a single high dose intraperitoneally (I.P.) of DEN, and after one week recovery period, the rats were administered with 2-AAF. Three doses of 2-AAF were orally administered on three alternative days among the first week of each month for entire study period (four months). Therefore, a total of 3 and 12 doses of 2-AAF were administered to the animals in the 1 M and 4M treated groups, respectively. The rats in control group received normal saline at the same schedule. The rats in the 1 M and 4 M groups were kept in a glass chamber containing cotton soaked with diethyl ether to be anesthetized and sacrificed at respectively one and four months after carcinogen treatment, respectively. At the time of sacrificing, the animals were perfused transcardially with saline and after their death they were dissected to excise livers for further analysis. 

### Histological examination 

Livers were fixed in 10% formalin, sliced, dried out and buried in paraffin. Cross-sections were taken and stained with Hematoxylin and Eosin. Sections were mounted with DPX mountant (Sigma-Aldrich, USA) and checked employing light microscope for histological changes. 

### Proteomic analysis of differentially expressed proteins

The Bradford’s method was used to measure protein concentration (7). Depletion of albumin in serum samples and their preparation, 2 dimensional (2D)-electrophoresis of the total serum proteins and their analysis with PD- Quest software and ultimately MALDI-TOF-MS/MS characterization were performed as previously described (8, 9). 

### Validation of protein expression by Western blot analysis 

30 µg of total serum protein was fractionated on 10% poly acrylamide gel at 80 V and it was transferred to polyvinylidene difluoride (PVDF) membrane employing Hoefer Western blotting apparatus (HoeferInc, USA, 4°C, 150 mA for three hours). Immunodetection was performed using 1:500 diluted primary antibody (Sigma-Aldrich, USA) in Tris-buffered saline (TBS) overnight at 4°C and 1:4000 diluted HRP conjugated anti-rabbit secondary antibody (Sigma-Aldrich, USA) for three hours. The protein expression was visualized with diaminobenzidine (DAB, Sigma-Aldrich, USA) and LuminataTM Forte Western HRP Substrate (Millipore, USA) system. Analyses of clinically approved liver cancer patients sera (including two male patients aged 35 and 73 years used for the analyses) and controls (including two matched age healthy males with liver cancer patients used for the analyses) were carried out after receiving the approval of Jamia Hamdard Institutional Ethics Committee (JHIEC). The informed consent was obtained from all participating subjects. The HCC patients were clinically approved and were under various therapies. 

### Statistical analysis

The experiments were carried out in triplicate and data are revealed as means ± standard error of the mean (SEM). The significance of differences (control vs. treated groups) was analyzed employing One-way ANOVA pursued by Dunnett test and they were considered statistically significant when P<0.05. 

## Results

### Development of hepatocellular carcinoma model and serum analysis of carcinogen treated rats and controls

**Fig.1:**
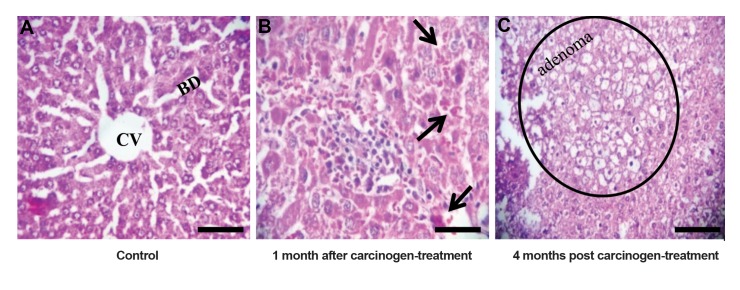
Histological analysis of liver tissue. Photomicrographs show histological changes in liver at high power. A. Normal architecture of central vein (CV) and bile duct (BD) are shown in the control liver (each treated group has its own control to be compared), B. One month post carcinogen treatment, inflammation and hemorrhage leading to HCC initiation are shown, using the arrows, and C. Development of adenoma (within the circle) was observed at four months aftercarcinogen treatment. Cystic degeneration of hepatocytes has been shown at tumors within the circle (scale bar: 20 µm at ×400 magnifications).

HCC was induced by administrating chemical carcinogens (DEN and 2-AAF) in male Wistar rats, as previously reported by us (6). Histological analysis revealed disease initiation at one month and development of cancer and tumorigenesis at four months after carcinogen treatment (Fig .1) (8). 

**Fig.2:**
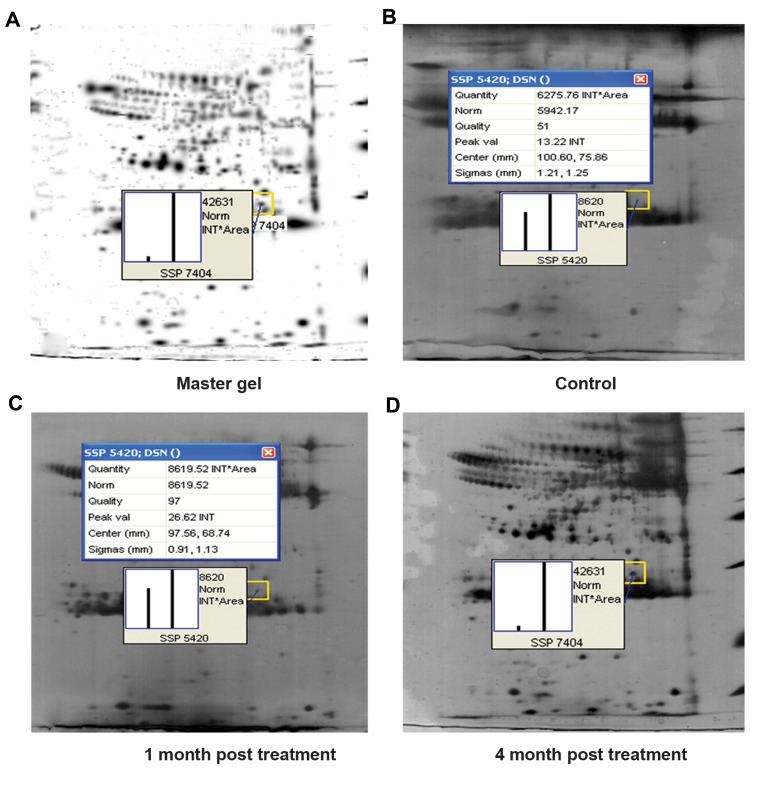
PD-Quest analysis of serum protein on 2D gels. The master gel represents protein spots from both control and treated groups, with pop up graph for proteinof interest. The protein expression has been quantified based on intensity (INT) × area. A. The protein of interest quantification for the SSP 7404 master gel hasbeen calculated based on the protein intensity of four months post carcinogen-treatment group. The protein spots were quantified and compared to B. SSP 5420 in control, C. SSP 5420 in one month post treatment groups, and D. SSP 7404 in the four months after carcinogen-treatment group. The expression intensity showedsignificant elevation of target protein level. SSP; Stands for sample spot protein and each spot protein has a unique SSP number.

**Fig.3:**
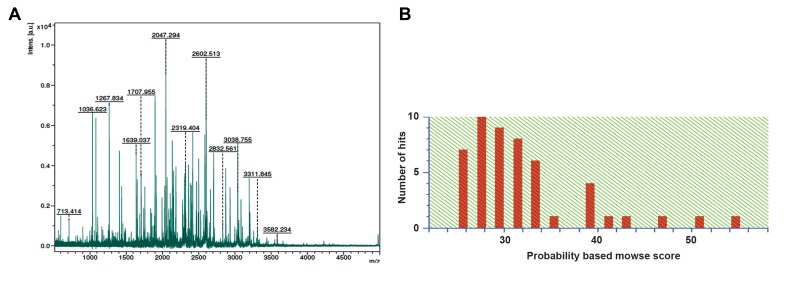
MALDI-TOF-MS/MS characterization of target spot. A. Spectra for the target spot were shown by MALDI-TOF-MS/MS characterization and B. MASOT histogram analysis: probability based on MOWSE score is defined as -10×log (P), where P is probability of the observed match with a random event. Individual protein scores, greater than 59, indicate identity or extensive homology (P<0.05).

Reproducible results were obtained following repeatedly performing 2D-electrophoresis analyses. The analyses of 2D gels using PD-Quest were assigned unique sample spot protein (SSP) numbers to protein spots and compared differentially expressed proteins (8-10). One of these proteins, up-regulating at one month (initiation stage of HCC) and four months (tumorigenesis stage) after carcinogen treatment, was selected for further analysis. Detailed expression analysis of this protein following carcinogen administration has been illustrated in Figure 2. Changes in the levels of protein expression were statistically significant (P<0.05). The protein spot was excised from 2D gels, digested and the mass fingerprinting of its peptides was obtained by MALDI-TOF-MS/ MS characterization. The protein was characterized as orthologue of cytosolic phospholipase A_2_ delta, gi|109470683, [*Rattus norvegicus*], using NCBI database search by MASCOT software (Fig .3A, B). Detailed analysis and characterization of cytosolic phospholipase A2 delta have been shown in Table 1.

**Table 1:** MALDI-TOF-MS/MS characterization of cytosolic phospholipase A_2_ delta


Observed	Mr (Expt)	Mr (Calc)	ppm	Start-End	Miss	Peptide

1094.6700	1093.6627	1093.5516	102	389-398	0	K.DLEGPISHAR.E
1267.8339	1266.8267	1266.6357	151	478-487	0	K.ENHLETLHFK.E
1639.0371	1638.0298	1637.8485	111	445-458	1	K.LHGQVTDQKLSEQR.A
1740.0199	1739.0126	1738.8348	102	291-305	0	R.LSYGLCPEEQAFLGR.R
1839.1123	1838.1051	1838.0513	29.3	309-325	1	K.LVAAALKQALQLDEDLK.E
1994.2330	1993.2257	1992.9840	121	689-706	1	K.GLQQSGKYCSAQGLPFPR.V
2047.2940	2046.2867	2046.0745	104	791-808	0	R.LSEYNIQNNQGTILQALK.T
2107.4134	2106.4061	2106.0700	160	180-199	1	R.AGSTTMAAGQDKLELELMLK.G
2121.3561	2120.3488	2120.0936	120	24-41	1	R.QEEASVFCQLTVKILEAR.S
2319.4037	2318.3964	2318.1430	109	132-151	0	K.TFSLNPQGPEELDVEFLVER.T
2324.3359	2323.3286	2323.1630	71.3	286-305	1	K.ELSVRLSYGLCPEEQAFLGR.R
2399.2732	2398.2659	2398.1490	48.8	502-522	1	K.YGGFVPSELFGSEFFMGRLMK.R
2418.4232	2417.4159	2417.1493	110	169-191	1	R.ELSHLDVSLDRAGSTTMAAGQDK.L+Oxidation (M)
2832.5611	2831.5539	2831.3775	62.3	357-381	0	K.LGLLDCVTYFSGISGATWTMAHLYR.D
3023.9028	3022.8955	3022.4634	143	755-781	1	R.SPDELKAGQVDLTGVASPYFLYNMTYK.N+Oxidation (M)
3257.9532	3256.9459	3256.6616	87.3	425-453	1	R.EEQGYTVTIADLWGLVLESKLHGQVTDQK.L


Mr; Average molecular mass of the peptide in kilodalton, Expt; Experimentally determined molecular mass, Calc; Theoretically calculated mass of peptide based on atomic mass of the component, and Ppm; Parts per million.

**Fig.4:**
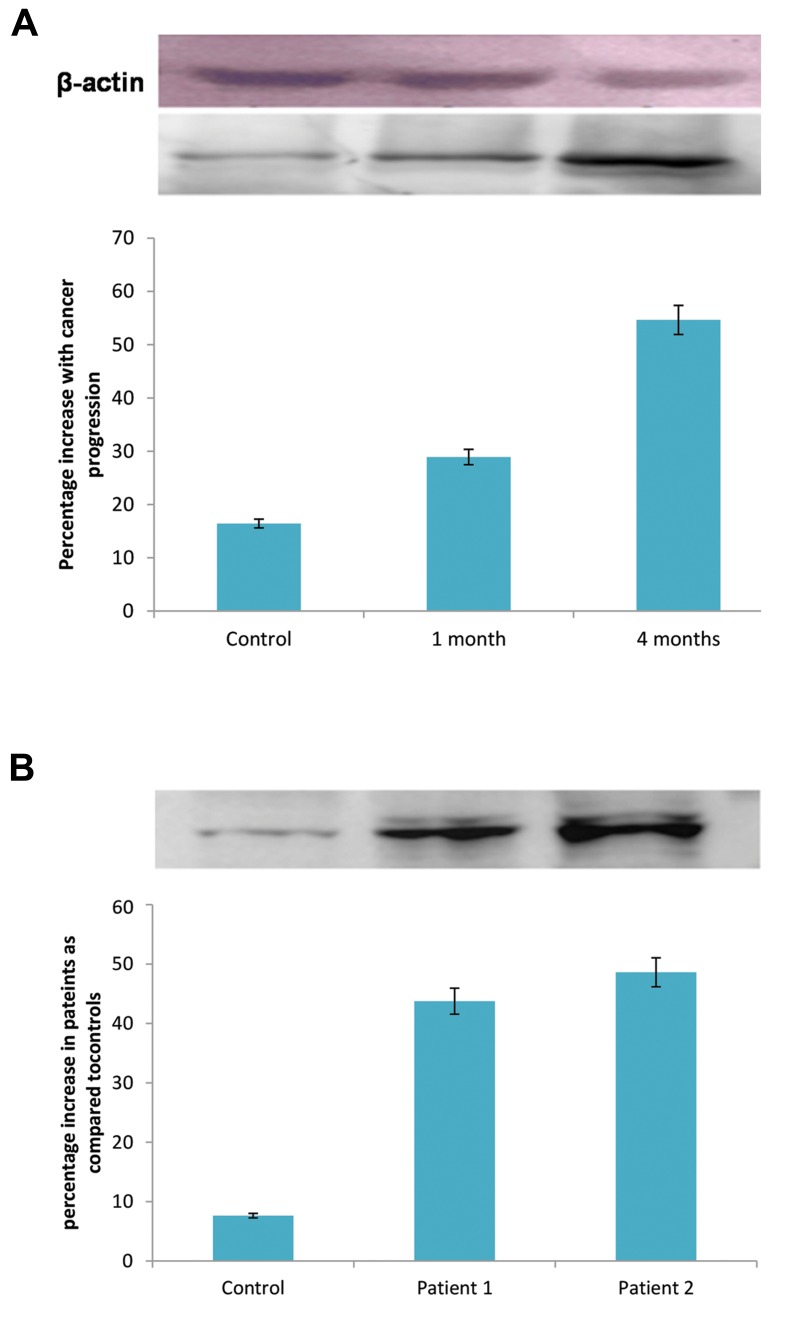
Expression analysis of cytosolic phospholipase A_2_ delta by Western blotting. A. ß-actin was used as internal control. Analyses showed time anddose dependent elevation in the expression of protein of interest, in ratsbelong to carcinogen treated groups, by liver cancer progression. Consistentincrease in protein expression has been shown during liver cancer progressionand B. Significant elevation in expression of cytosolic phospholipase A_2_ delta has been shown in sera of clinically confirmed liver cancer patients ascompared to the healthy controls (P<0.001, n=3, serum samples were takenfrom two male patients at age 35 and 73 for the analyses. Sear of healthycontrol was drawn from the same age group). The Image j software wasused to quantify the intensity of protein bands. Fold change in expressionof cytosolic phospholipase A_2_ delta was normalized over the age-matchedcontrols. Data are presented as mean ± SEM (n=3), using one way ANOVAfollowed by Dunnett test.

Western blot analysis revealed up-regulation of cytosolic phospholipase A_2_ delta expression in the serum of carcinogen treated rats vis-a-vis age-matched controls. Expression of cytosolic phospholipase A_2_ delta was elevated at one month after carcinogen treatment and it was continued to increase during cancer progression until tumor stage at four months post carcinogen treatment. Expression of ß-actin was considered as internal control (Fig .4A).

Moreover, Western blot analyses of clinically confirmed liver cancer patients’ sera showed elevation of cytosolic phospholipase A_2_ delta expression compared to the controls (Fig .4B). This observation validated our experimental results. 

## Discussion

Neoplastic cell induction served as implication of cancer initiation in liver tissue of rats. Our method for animal model development is novel, as it neither requires carcinogen doses causing necrosis nor partial hepatectomy (6, 10). The serum protein profile of carcinogen treated rats and controls were compared and differentially expressed proteins were identified. However, one of these proteins was further characterized as cytosolic phospholipase A_2_delta. Changes taking place in the expression of HCC- related proteins have been systematically monitored during various stages of HCC development, from the initiation of cancer to hepatotumorigenesis, when fully grown tumors were observed. 

The importance of cytosolic phospholipase Aenzymes in cancer progression is of the considerable interests, as these enzymes play important role in the pathways associated with progression of cancer. This enzyme family controls cell proliferation, differentiation, survival and motility in almost all tissues. Their increased expression results in dysregulation and facilitates unlimited growth of tumors and metastasis for cancer cells. Significant role of this family members has been implicated in tumor progression and tumorigenesis (11). This family is composed of six intracellular enzymes simply indicated as cytosolic phospholipase A_2_-α, -β, -γ, -δ, -ε and –ζ (12). Cytosolic phospholipase A_2_ family mediates biologically active fatty acids release from the pool of phospholipids located in membranes (13) of virtually all cells in humans and rodents (12). Aberrant expression of cytosolic phospholipase A_2_ family has been linked to progression of malignancies such as prostate, liver (13) and colon cancers (14). A study has reported that elevating cytosolic phospholipase A_2_ expression has been taken place through pathways associated with ERK1/2 and p38 MAPK. The study reported that cytosolic phospholipase A_2_ expression is significantly associated with vascular endothelial growth factor expression; however, its expression was not related to any clinico-pathological specification (14). No much information is available about the function of cytosolic phospholipase A_2_ family *in vivo* (12). Among three main classes of cytosolic phospholipase A_2_ family in mammals, cytosolic phospholipase A_2_-α has gained the most attention, regarding that is widely expressed in virtually all mammalian cells (14). Cytosolic phospholipase A_2_-α is activated by transforming growth factor beta (TGF-ß) regulating growth of primary and transformed hepatocytes. The inter relationship among cytosolic phospholipase A_2_-α and TGF-ß signaling pathways has been reported in primary hepatocytes of rats and human HCC; thus cytosolic phospholipase A_2_-α is an important factor regulating TGF-ß signaling pathway and controlling proliferation of hepatocytes and hepatocarcinogenesis (15). Cytosolic phospholipase A_2_-α regulates biosynthesis of prostaglandins through arachidonic acid cleavage, from membrane phospholipids (12), through cyclooxigenase (COX) (16). The prostaglandins increase storage of triglycerides in hepatocytes leading to liver damage and cirrhosis (12). This pathway is activated in variety of cancers including HCC (15). Arachidonic acid, as a substrate for COXs and lipoxygenases (14), is a necessary factor that producing bioactive eicosanoids and platelet activating factor which, in turn, regulate inflammation (17), tumor cell proliferation and motility, differentiation, survival, invasion, angiogenesis and metastasis in HCC (13, 15). We observed elevated levels of cytosolic phospholipase A_2_ delta in the serum of HCC rats and in human patients with liver cancer. This suggests that is one of the important factors associated with HCC initiation and progression leading to hepatotumorigenesis. Elevation of cytosolic phospholipase A_2_ delta expression in liver cancer might be associated with dysregulation of lipid metabolism and liver damage, causing cancer initiation in tissue at precancerous stage, while the epithelial cells are actively proliferating.

## Conclusion

Taken together, the present study suggests that evaluation of cytosolic phospholipase A_2_ delta concentration, alone or in consolidation with other conventional markers, may provide critical knowledge for the early noninvasive disclosure of HCC. Moreover, cytosolic phospholipase A_2_ delta might also be served as a potential target to find out the status and progression of liver cancer. 
